# ATR–FTIR Spectral Analysis and Soluble Components of PM_10_ And PM_2.5_ Particulate Matter over the Urban Area of Palermo (Italy) during Normal Days and Saharan Events

**DOI:** 10.3390/ijerph16142507

**Published:** 2019-07-13

**Authors:** Daniela Varrica, Elisa Tamburo, Marcello Vultaggio, Ida Di Carlo

**Affiliations:** 1 Dipartimento Scienze della Terra e del Mare (DiSTeM), Via Archirafi 22, 90123 Palermo, Italy; 2 Risorse Ambiente Palermo (RAP), Piazzetta B. Cairoli, 90123 Palermo, Italy; 3 CNRS/INSU-Université d’Orléans—BRGM, UMR 7327, Institut des Sciences de la Terre d’Orléans, 1A rue de la Férollerie, 45071 Orléans, France

**Keywords:** particulate matter, PM_10_ and PM_2.5_, ATR-FTIR, ionic soluble components, saharan dust events, sirocco winds

## Abstract

Several epidemiological studies have shown a close relationship between the mass of particulate matter (PM) and its effects on human health. This study reports the identification of inorganic and organic components by attenuated total reflectance-Fourier-transform infrared spectroscopy (ATR-FTIR) analysis in PM_10_ and PM_2.5_ filters collected from three air quality monitoring stations in the city of Palermo (Sicily, Italy) during non-Saharan dust events and Saharan events. It also provides information on the abundance and types of water-soluble species. ATR-FTIR analysis identified sulfate, ammonium, nitrate, and carbonate matter characterized by vibrational frequencies at 603, 615, 670, and 1100 cm^–1^ (SO_4_^2–^); at 1414 cm^–1^ (NH_4_^+^); at 825 and 1356 cm^–1^ (NO_3_^–^); and at 713, 730, and 877 cm^–1^ (CO_3_^2–^) in PM_10_ and PM_2.5_ filters. Moreover, aliphatic hydrocarbons were identified in the collected spectra. Stretching frequencies at 2950 cm^–1^ were assigned to CH_3_ aliphatic carbon stretching absorptions, while frequencies at 2924 and 2850 cm^–1^ indicated CH_2_ bonds. In filters collected during Saharan dust events, the analysis also showed the presence of absorbance peaks typical of clay minerals. The measurement of soluble components confirmed the presence of a geogenic component (marine spray and local rocks) and secondary particles ((NH_4_)_2_SO_4_, NH_4_NO_3_) in the PM filters. ATR-FTIR characterization of solid surfaces is a powerful analytical technique for identifying inorganic and organic compounds in samples of particulate matter.

## 1. Introduction

The urban air people breathe contains several solid and gaseous chemicals that have significant negative effects on public health [1,2,3]. Several epidemiological studies have shown a close relationship between air pollution and various respiratory tract diseases (allergies, asthma, lung emphysema), lung cancer, and cardiopulmonary mortality, which commonly affect urban populations [4,5,6,7,8,9]. The World Health Organization (WHO) [10] and the Directive of the European Parliament [11] established that daily values in Europe for concentrations of particulate matter with sizes of ≤10 and ≤2.5 µm (i.e., PM_10_ and PM_2.5_) should not exceed 50 µg/m^3^ and 25 µg/m^3^, respectively. Particulate matter with a size ≤10 µm is considered to be particularly detrimental to human health, but the nature of the PM is equally crucial as particle types have highly variable toxicity levels. Particulate matter comprises a range of particles such as mineral dust, metals, metalloids, sea salts, ammonium nitrate and sulfate, organic compounds, and elemental carbon. The abundance of the various organic and inorganic components is temporally and spatially variable [12]. Some are directly emitted into the atmosphere by either natural or anthropogenic sources (primary particles), while others are the result of homogeneous or heterogeneous nucleation and condensation of gaseous precursors (secondary particles). The Mediterranean area is often affected by Saharan dust events, which increase PM_10_ and PM_2.5_ concentrations beyond European recommended values, mainly in southern Europe. Saharan dust is a mixture of mineral particles (quartz, calcite, dolomite, and clay minerals) and organic matter [13]. Some studies have suggested that Saharan dust has a significantly negative impact on air quality, visibility, and human health [14,15,16]. Several authors described increased asthma, rhinitis, cardiovascular disease, and mortality [17,18]. Other authors found no association between dust events and hospitalizations [19,20,21,22,23], increased mortality, or increased potential oxidative water-soluble fractions in PM_10_ and PM_2.5_ [24] compared to anthropogenic dust. 

Water-soluble components (WSCs) are among the main components of total particulate matter [25,26], typically contributing about 50%–70% of the weight. WSCs are associated with degraded atmospheric visibility and adverse effects on human health [27,28,29]—they also contribute to the formation of acid rain, which promotes the faster decay of buildings. The main analytical technique used to determine water-soluble components is ion chromatography (IC). In recent years, Fourier-transform-infrared spectroscopy (FTIR) has become important in identifying aerosol composition and quantifying the mass of organic and inorganic compounds in particulate matter [30,31,32,33]. FTIR coupled with accessories like attenuated total reflectance (ATR) allows the analysis of a wide range of solid and liquid components [34]. 

In this study, we present data on the chemical composition of water-soluble components in PM_10_ and PM_2.5_ samples collected in an urban area of southern Italy. The city of Palermo, chosen for our case study, is affected by urban pollution and natural particulate matter from a range of sources. The principal sources in the study area are gasoline- and diesel-powered vehicles, an active commercial and tourist harbor, domestic heating, and a geogenic component that includes soil erosion, marine aerosol, and sporadic Saharan dust events. The aim of this paper is to identify the principal functional groups of inorganic and organic components in atmospheric aerosols by ATR-FTIR analysis. Moreover, we report the results of FTIR analysis carried out on samples of PM_10_ and PM_2.5_ filters taken during Saharan dust events that affected the Mediterranean area. 

## 2. Materials and Methods 

### 2.1. Site Details

**Figure 1:**
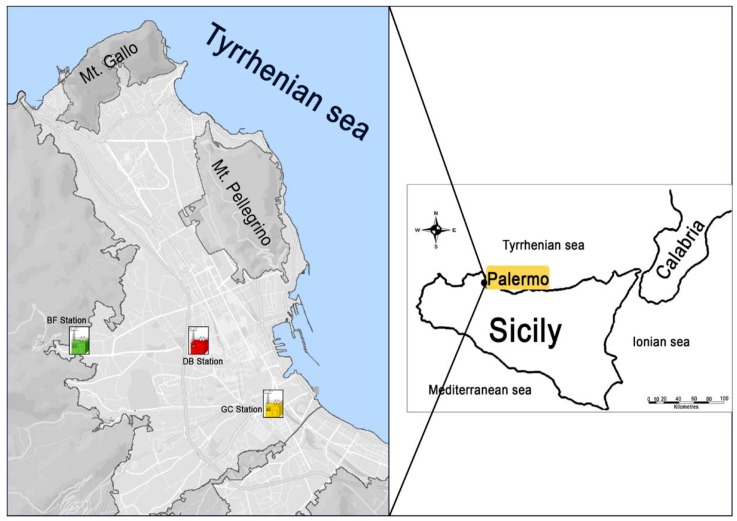
Location of study area with sampling sites. BF, Boccadifalco; DB, Di Blasi; GC, Giulio Cesare.

Palermo is the largest urban area of Sicily, with about 680,000 inhabitants and a metropolitan area populated by more than 1 million people. The city is situated on the north-western coast of the island, bordered on the northeast by the Tyrrhenian Sea and surrounded by mountains (Monti di Palermo) reaching 500–1000 m above sea level (Figure 1). 

**Figure 2:**
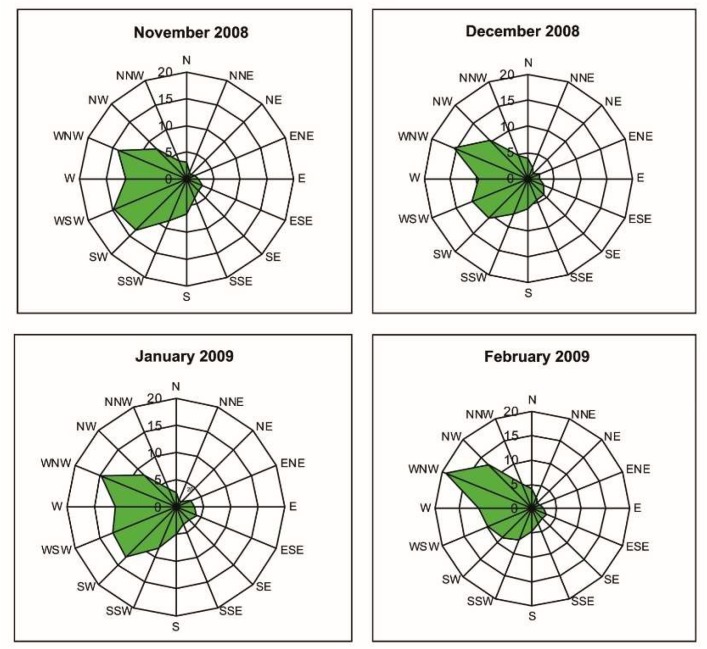
Prevailing winds at Palermo during the sampling period.

The study area is entirely covered by sedimentary rocks (limestone, clay, marly-clay, and white or yellow quaternary biocalcarenite). The climate of Palermo is typically Mediterranean, with hot summers and temperate winters. Among the stations studied, only Boccadifalco (BF) station records weather data representative of the entire agglomeration where the other stations of the present study (Giulio Cesare (GC) and Di Blasi (DB) are located. Figure 2 shows the wind rose of the sampling period (November 2008–February 2009). From the monthly wind roses during the winter months, the prevailing wind direction is from the WNW and WSW sectors. In the same period, close to 5% wind direction from the S and SSE sectors (Sirocco winds) has been registered. During autumn and spring in the city, there are frequent warm winds coming from south-east (Sirocco winds) carrying dust raised from the Sahara Desert region throughout the Mediterranean basin. Over the sampling period, the weather monitoring station located in the peripheral area of Palermo (BF station) registered six periods of 1–2 days of Saharan dust intrusions.

### 2.2. Sampling Sites

A total of 348 daily samples, 308 PM_10_ and 40 PM_2.5_, were collected from November 2008 to February 2009. To meet the requirements of Directive 1999/30/EC (EU Commission, 1999), PM_10_ sampling was performed according to European Standard EN12341 (CEN, 1998), with a low-volume system equipped with a sampling inlet head (Zambelli Explorer Plus Controller 16) operating at a constant sampling rate (2.3 m^3^h^-1^). Particles were collected on standard 47 mm quartz filters (Advantec, grade QR100). The sampling time was 24 h, from midnight to midnight. PM_2.5_ sampling was performed according to European standard EN 14907 (CEN 2005). At Di Blasi (DB) station, simultaneous sampling of PM_10_ and PM_2.5_ was carried out. PM_10_ mass determination was performed by β-ray attenuation method, model Environment MP101M.C (CNR–Italy certified). The beta attenuation instrument is compliant with EN 12341 for PM_10_ and is approved as federal equivalent method by US the Environmental Protection Agency for PM_10_. The detection is done every 2 hours (12 detections in 24 hours). Initial and final weighing of PM_10_ and PM_2.5_ filters were carried out in a temperature- and humidity-controlled room (T = 20 ± 1 °C, RH = 50 ± 5%) after the filters had been conditioned for 48 h before and after sampling. Three air quality monitoring stations belonging to the municipal monitoring network (RAP-ex AMIA) were chosen for this study (Figure 1). 

The Di Blasi (DB) station is located close to a crossroads with traffic lights at pedestrian crossings and is characterized by high traffic flow, consisting of cars, heavy-duty vehicles, and buses. Giulio Cesare (GC) station is situated in a large square in front of the railway station, exposed to heavy traffic composed of cars as well as urban and regional buses. The Boccadifalco (BF) station is a suburban background station, situated leeward of the sea breeze, without any direct influence of urban activities. It has lower traffic density than the other stations and was selected as a control site to monitor the hypothetical background level of pollution. Filters used for analysis were selected based on the simultaneity of daily sampling between the three monitoring stations. ATR-FTIR spectroscopy was used to analyze 13 PM_10_ filters from the suburban background station (BF), 36 PM_10_ filters from the urban station (GC), 40 PM_2.5_ filters from the urban station (DB), and one composite sample of Saharan dust deposited in Palermo town. A total of 1 g of Saharan dust was taken near GC station using a plastic brush and tray and stored in plastic bags. The sample was initially sieved through a 63 μm sieve to remove coarse components. Afterward, screening through a 20 μm mesh sieve was necessary to obtain a finer fraction for FTIR analysis. The following were analyzed for water-soluble ions: 13 PM_10_ filters from BF station, 30 PM_10_ filters from GC station and 30 PM_2.5_ filters from DB station.

### 2.3. Analytical Procedures

#### 2.3.1. FTIR Spectra 

A Bruker Optics (Tensor 27) IR (Bruker Corporation, Billerica, MA, USA) spectrometer equipped with a deuterated triglyceride sulfate detector was operated with Opus software from Bruker to obtain the spectra of ambient air samples. An ATR accessory with a germanium crystal flat plate was coupled with the spectrometer for data acquisition. Aerosol sample spectra were obtained over wavelengths between 4000 and 400 cm^–1^ (mid-infrared region) with 2 cm^–1^ resolution by averaging 32 scans. Each aerosol sample was scanned by placing the quartz fiber filter sample-side down on the ATR crystal and applying the pressure tower. Each IR spectrum was corrected for optical effects with the ATR correction algorithm in Opus. A blank quartz fiber spectrum was obtained with each set of daily samples to account for any changes in the absorbance bands due to instrument drift. Between each sample spectrum acquisition, the ATR crystal was cleaned with ethanol, and an air background spectrum was obtained. The FTIR operation method is explained in Doyle [35] and Simonescu [36].

#### 2.3.2. Water-Soluble Ions

Water-soluble ions were extracted from filter samples with 20 mL ultra-pure Milli-Q (Merck Millipore, Burlington, MA, USA) water (18MΩ cm) and shaken for 24h. The extracts were filtered through a 0.45 µm pore size polytetrafluoroethylene filter (Sartorius) and then stored in sterile 50 mL polypropylene centrifuge tubes. Each extract was analyzed the day after the extraction procedure for Ca^2+^, Mg^2+^, Na^+^, K^+^, Cl^–^, SO_4_^2–^, and NO_3_^–^ ions by ion chromatography (Dionex 100), with precision better than ± 5%. Cations were measured using a Dionex IonPac CS12A (Thermo Fisher Scientific, Waltham, MA, USA) column with 20 mM methanesulfonic acid as the eluent. Anions were measured using a Dionex IonPac AS14 (Thermo Fisher Scientific, Waltham, MA, USA) with 3.5 Mm Na_2_CO_3_ and 1.0 mM NaHCO_3_ as the eluent. The limit of detection was evaluated by solution extracts for three blank filters in 0.02–0.05 and 0.04–0.05 mg/L for cations and anions, respectively. NH_4_^+^ ions were determined spectrophotometrically at λ = 420 nm (Thermo Scientific Evolution 600) using Nessler’s reagent (0.09 mol/L solution of potassium tetraiodomercurate (II) (K_2_[HgI_4_]) in 2.5 mol/L potassium hydroxide). The ion chromatograph operation method is explained in Michalski [37]. 

## 3. Results and Discussion

### 3.1. Mass Levels of PM_10_ and PM_2.5_

Table 1 shows the mass levels of PM_10_ at the urban and peripheral stations (GC, DB, and BF) and of PM_2.5_ at the urban DB station. 

The mean PM_2.5_ and PM_10_ concentrations fall within the range reported for European urban areas [38,39,40]. The highest mean PM_10_ value was observed at the urban DB station (42 µg/m^3^), one of the most heavily traffic-exposed sites in Palermo, followed by GC station (39 µg/m^3^) and BF station (16 µg/m^3^). The average PM_2.5_ value observed at DB station is 29 µg/m^3^. For the 112 days analyzed (November 2008–February 2009), Saharan dust events influenced mass levels in the Palermo atmosphere on about 6% of the days. During these events, concentrations of 130–158 µg/m^3^ and 78 µg/m^3^ were measured for PM_10_ and PM_2.5_, respectively. In Palermo in February 2009, PM_2.5_ reached a daily concentration of 120 µg/m^3^ and PM_10_ reached values between 220 and 276 µg/m^3^ following a high-intensity Saharan dust event. The value of PM_2.5_ is comparable to that found by Remoundaki et al. [41] in Athens in February 2009 (100 µg/m^3^). During other, less-intense Saharan dust events, PM_2.5_ concentrations (average 62 µg/m^3^) increased by 50% and PM_10_ (average 86–119 µg/m^3^) by 65–80%. The values reported are higher than those published in previous studies concerning southern Italy (Rome: PM_2.5_, 25.6 µg/m^3^; PM_10_, 47.2 µg/m^3^ [16]; Salento: PM_2.5_, 36.6 µg/m^3^; PM_10_, 137 µg/m^3^ [24]; Bari: PM_2.5_, 31–49 µg/m^3^; PM_10_, 50–71 µg/m^3^ [42]), but the higher percentages during Saharan dust events are comparable [24,43,44,45]. In general, the contribution of particulate matter is evidenced in coarse rather than fine fractions [41]. The influence of Saharan dust on the Mediterranean basin has been estimated to be about 10–20% per year, thus many European countries have exceeded the PM limits recommended by the European Directive. The PM_2.5_/PM_10_ ratio has been widely used in environmental studies as an indicator of the contribution from stationary vs. mobile source emissions to the environment. 

The average PM_2.5_/PM_10_ ratio measured at DB station is 0.70. This value is typical of urban environments with high traffic density [26,40,46,47,48,49]. During Saharan events, the PM_2.5_/PM_10_ ratio was only 0.58, indicating a greater natural contribution of coarse than fine particles.

### 3.2. Spectral Analysis

**Figure 3:**
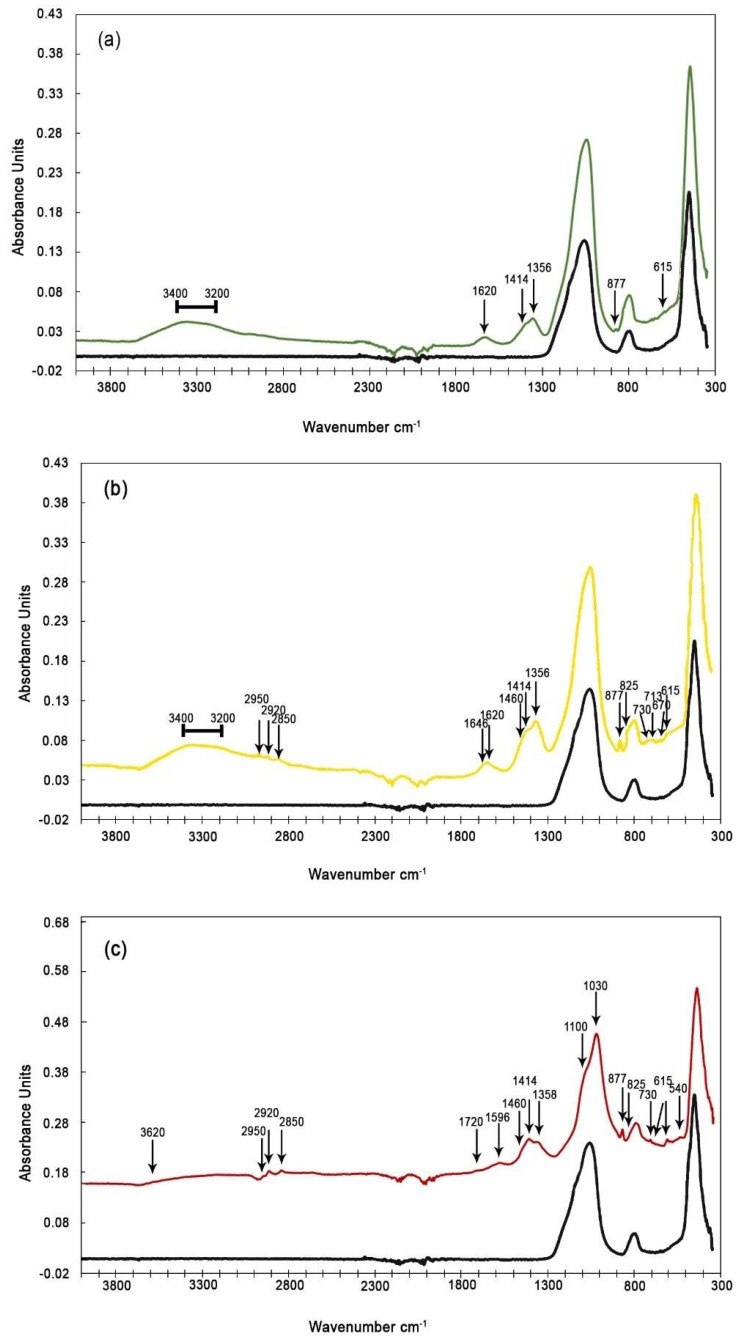
FTIR spectra of (**a**) suburban PM_10_ filter (BF station); (**b**) urban PM_10_ filter (GC station); and (**c**) urban PM_2.5_ filter (DB station). For each spectrum, we also report the spectrum of a blank quartz filter (black line) for comparison.

The ATR spectra of PM_10_ and PM_2.5_ are shown in Figure 3a–c. The spectra identify different inorganic and organic molecules (Table 2). 

Some peaks have no well-defined forms, and the presence of a shoulder indicates that there are overlapping peaks due to several different types of molecules absorbing IR radiation within the same range. From comparing the spectra, it is observed that the samples are dominated by inorganic components common to all stations.

In PM_10_ and PM_2.5_ filters, we observed vibrational frequencies typical for sulfate, ammonium, nitrate, and carbonate ions (603, 615, 670, and 1100 cm^–1^ (SO_4_^2–^); 1414 cm^–1^ (NH_4_^+^); 825 and, 1356 cm^–1^ (NO_3_^–^); and 713, 730, and 877 cm^–1^ (CO_3_^2–^). The presence of (NH_4_)_2_SO_4_ and NH_4_NO_3_ compounds is supported by spectra shown in Figure 3b,c revealing absorption frequencies at 825 and 1356 cm^–1^ (group NO_3_^–^), at 615 and 1100 cm^–1^ (group SO_4_^2–^), and at 1414 cm^–1^ (NH^4+^ ion) [30,50]. The inferred 1100 cm^–1^ peak is a shoulder of the peak observed at 1060 cm^–1^. The 1100 cm^–1^ peak is assigned to the v3 asymmetrical stretching vibration of sulfate ion [51,52].

In all spectra (for coarse and fine particles), absorption frequencies at 1620 cm^–1^ and in the range of 3240–3400 cm^–1^ have been detected that can be attributed to O–H stretching, indicating crystalline water in gypsum [34,53].

The presence of CaSO_4_ × 2H_2_O is more evident in coarse than fine particles. The common presence of CaSO_4_ × 2H_2_O signals supports the widely accepted hypothesis that the sulfation process is important in urban environments [54]. Varrica et al. [55] observed CaSO_4_ × 2H_2_O crust on CaCO_3_ particles by scanning electron microscopy (SEM) of samples also collected in Palermo. In samples of “black crust” formed on historical buildings in Palermo, Montana et al. [56] determined δ^34^S values ranging from –0.5 to +5.0‰ (vs. Vienna Cañon Diablo Troilite (VCDT) scale), which suggests that most of the sulfur was derived from fossil fuel combustion. Moreover, Cesari et al. [43] observed that during Saharan dust events, the dominant form of sulfate is calcium sulfate rather than (NH_4_)_2_SO_4_. The absorption peaks at 713, 730, and 877 cm^–1^ are typical for the CO_3_ group [53,57,58], and the FTIR analysis of pure crystalline calcite and dolomite confirms that these peaks are related to CaCO_3_. The peaks of halite between 1000 and 1200 cm^–1^ are not clearly visible due to the absorption linked with the quartz filter. In the GC and BF samples, there is also a peak at 1620 cm^–1^ that can be attributed to one of the peaks of halite; at 1414 cm^–1^ the peak of halite is not visible as it is very small and overlaps with the ammonium ion peak.

Organic compounds are identified in the coarse and fine particle fractions of the urban stations but are absent from the filters collected at the suburban station. The aliphatic hydrocarbons (2850, 2920, and 2950 cm^–1^) were clearly identified in the collected spectra (Figure 3b,c) [30,31,34,59,60,61]. The stretching frequency at 2950 cm^–1^ is assigned to CH_3_ aliphatic carbon stretching absorption, while the frequencies at 2924 and 2850 cm^–1^ are due to CH_2_ bonds. An absorption peak at 1460 cm^–1^ comprises contributions from bending of CH_3_ and CH_2_ aliphatic carbon bonds [30,59]. Vibration around 1460 cm^–1^ is a shoulder of the peak at 1414 cm^–1^. The spectra for PM_2.5_ filters also show an absorbance peak at 1596 cm^–1^, identified as a C = C group [59,61]. The identification of other peaks for C = C aromatic group (1463–1511–1596 cm^–1^) is complicated by overlapping peaks due to several different types of molecules that absorb IR radiation within the same range.

### 3.3. Spectral Analysis Of Samples Collected During Saharan Dust Episodes

**Figure 4:**
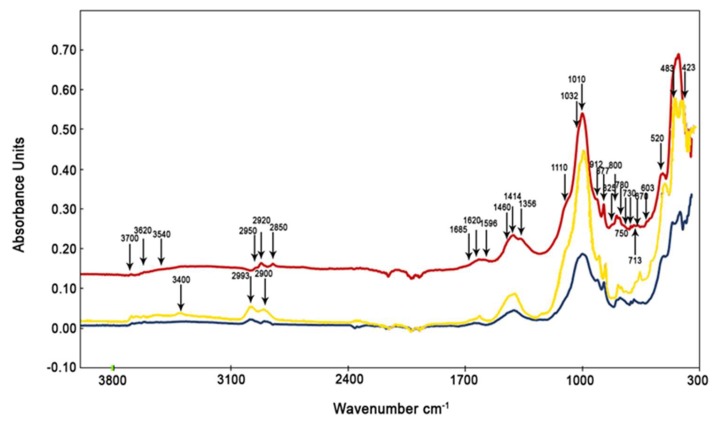
FTIR spectra of urban PM_10_ (GC, yellow line) and PM_2.5_ (DB, red line) filters. Blue line is FTIR spectrum of Saharan dust deposited in Palermo.

The particulate matter collected during Saharan dust events show peaks belonging to a group of clay minerals, which were not detected during non-Saharan events. Figure 4 shows the ATR spectra of the urban area (GC) PM_10_ filter, the urban area (DB) PM_2.5_ filter, and the Saharan dust.

Peaks at wavenumbers of 423, 463, and 520 cm^–1^ are associated with the O–Si–O bending of palygorskite and illite (426, 468, and 525 cm^–1^) [63]. The peak at 750 cm^–1^ identifies the inner layer vibration of Al–O–Si groups in illite [63,64]. Previous studies assigned the peak at 912 cm^–1^ to the deformation of Al–Al–OH groups in the dioctahedral layer of palygorskite [62,65]. The identification of kaolinite is characterized by the presence of peaks at 1010, 1032, and 1114 cm^–1^, representing the Si–O stretching group [67]. Peaks at 1032 and 1114 cm^–1^ are not distinct because they simultaneously characterize various molecules that vibrate in the same IR intervals, creating peak overlaps. 

Peaks at 3260, 3400, 3620, 3669, and 3695 cm^–1^ are all linked to the vibration of –OH groups belonging to different clay minerals. Peaks at 3260 and 3400 cm^–1^ are reported to relate to water stretching in palygorskite [63,65]. The OH groups located between tetrahedral and octahedral sheets are characterized by absorption near 3620 cm^–1^ in all clay minerals. They reside at the octahedral surface of the layers, forming weak hydrogen bonds with the oxygens of the Si–O–Si bonds on the lower surface of the next layer. A strong band at 3695 cm^–^^1^ relates to the in-phase symmetric stretching vibration. Weak absorptions at 3669 cm^–^^1^ are assigned to out-of-plane stretching vibrations [68,69]. In these samples, we found the same organic components as observed in the samples taken during non-Saharan events.

### 3.4. Water-Soluble Ions

Table 3 shows the mean concentrations of soluble components of PM_10_ and PM_2.5_ filters. Inorganic ions represent about 50%–70% of the total mass of PM_10_ and PM_2.5_. 

About 60% and 70% of total ions analyzed in PM_10_ and PM_2.5_ filters, respectively, are made up of NH_4_^+^, NO_3_^–^, and SO_4_^2–^. For urban stations (PM_10_ and PM_2.5_) the ammonium and calcium ions (expressed in neq/m^3^) are the most abundant cations. Magnesium and potassium are less abundant, contributing only about 1% to the total content of particulate matter. If the soluble calcium is derived from the alteration of carbonate rocks, a geogenic contribution of 10–14% of the total mass of the PM_10_ fraction from urban and suburban stations is estimated. In the fine PM_2.5_ fraction, a geogenic contribution is estimated to account for 9% of the total mass at the urban station.

**Figure 5:**
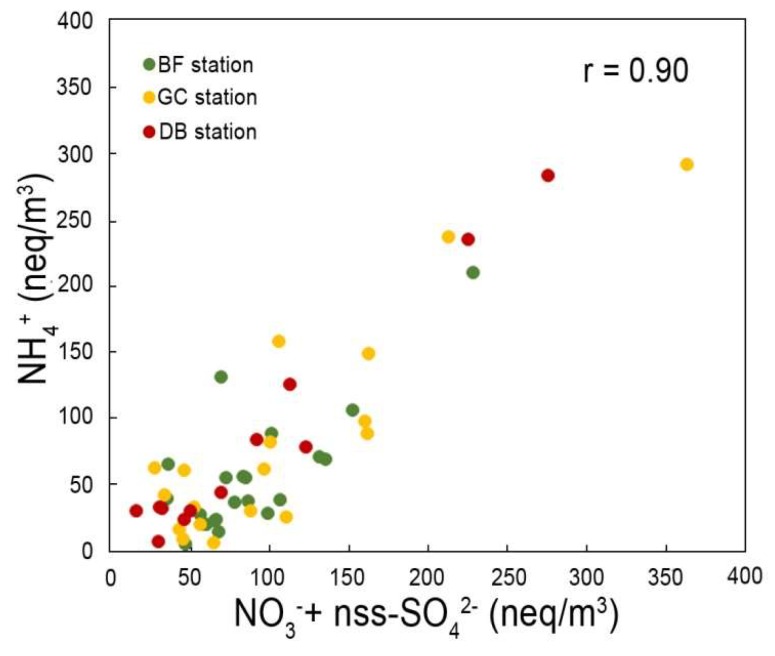
Plot of NO_3_^-^+nss–SO_4_^2-^ vs NH_4_^+^ ion concentrations. Data expressed in neq/m^3^.

NO_3_^–^ and SO_4_^2–^ anions have the highest concentration at all stations. In this study, the contribution of marine sulfate was calculated to have been around 6–10% in PM_10_ and 3% in PM_2.5_ fractions. The main source of SO_4_^2–^ in the atmosphere is from gas-to-particle conversion of SO_2_. NO_3_^–^ ions derive from the reaction of hydroxyl radicals, formed by photolysis of ozone molecules, with NO_x_ emitted by fossil fuel combustion. High concentrations of ammonium, sulfate, and nitrate ions demonstrate their involvement in secondary particulate formation. A significant correlation between NH_4_^+^ and (nssSO_4_^2–^ + NO_3_^–^) has been found (r = 0.90, *p* < 0.05; Figure 5), confirming the formation of ammonium sulfate and nitrate following neutralization of aerosol through heterogeneous atmospheric chemical reactions [47,70,71,72]. 

These sequences of reactions are strongly influenced by ambient temperature, relative humidity conditions, incidence of solar radiation, and above all the concentration of primary gases [73]. The equivalent ratio of NH_4_^+^/nss–SO_4_^2–^ in urban PM_10_ and PM_2.5_ is more than 1.5, characterizing the ambient atmosphere as ammonium-rich [74]. Nevertheless, as Figure 5 shows, the concentration of ammonium ions is insufficient to completely neutralize H_2_SO_4_ and HNO_3_. Total neutralization of the acid species is linked to the presence of carbonate rocks, abundant in the study area. The highest chlorine and sodium contents found in coarse samples (GC and BF) range between 1.70 and 1.19 µg/m^3^ and 1.14 and 1.45 µg/m^3^, respectively. The main source of Cl^–^ and Na^+^ in the study area is marine spray, accounting for 11–15% of the total mass in the PM_10_ fraction from urban and suburban stations. For fine PM_2.5_ fraction, sea salt contribution is estimated to account for 9% of the total mass at DB station. The average Na/Cl equivalent ratio measured in the PM_10_ and PM_2.5_ filters ranges between 1.4 and 1.8. These values are higher than those of seawater (0.85) and halite (1.0), suggesting a loss of chlorine ions due to chemical reactions that involve NaCl and HNO_3_ or H_2_SO_4_, bringing the formation of NaNO_3_ or Na_2_SO_4_ and gaseous HCl [66]. Similarly, a deficit of ammonium with respect to the collective concentration of SO_4_^2+^ and NO_3_^–^ (neq/m^3^) suggests that a proportion of these ions is lost via formation of NH_4_Cl or HCl and NH_3_ [47,51]. 

## 4. Conclusions

The main objective of this study was to verify the potential of ATR-FTIR to identify organic and inorganic groups present in PM_10_ and PM_2.5_. The use of ATR-FTIR led to the identification of absorption bands characteristic of sulfate, ammonium, nitrate, and carbonate by vibrational frequencies at 603, 615, 670, and 1100 cm^–1^ for SO_4_^2–-^, at 1414 cm^–1^ for NH_4_^+^, at 825 and 1356 cm^–1^ for NO_3_^–^, and at 713, 730, and 877 cm^–1^ for CO_3_^2–^ common to all filter types (PM_10_ and PM_2.5_). Vibration frequencies at 1620 cm^–1^ and in the range of 3240–3400 cm^–1^ indicate O–H stretching of crystalline water in gypsum. The presence of gypsum in the particulate matter of Palermo confirms the hypothesis that sulfation processes play an important role in urban areas. Moreover, in urban spectra, several organic compounds were identified, while aliphatic compounds were not detected at the suburban station. The ATR-FTIR analysis of filters taken during Saharan dust events shows the presence of absorbance peaks typical for clay minerals. The minerals found were palygorskite, illite, and kaolinite, which are typical for Saharan desert environments.

The water-soluble components represent about 50%–70% of the total mass of PM_10_ and PM_2.5_. Nitrate and sulfate ions had the highest concentrations at all stations, confirming their involvement in secondary particulate formation. The results show that ammonium ions are not able to neutralize most of the nitric and sulfuric acids present in aerosols. The main geogenic sources in the study area are marine spray and local rocks. 

The data of this study shows that ATR-FTIR, used here as a qualitative approach, is a powerful analytical technique for the identification of inorganic and organic compounds in PM_10_ and PM_2.5_ filters. Moreover, the simplicity of the substrate preparation, the excellent reproducibility of the results, the non-destruction of the sample, and above all the fast identification of components of particulate matter confirm the opportunity to use this analytical technique for qualitative analysis, and to characterize variations in the chemical composition of aerosol particles during intense pollution episodes. 

## Figures and Tables

**Table 1:** Characteristics of PM_10_ and PM_2.5_ samples at the three monitoring stations during non-Saharan dust events and Saharan dust events. Mass values expressed in µg/m^3^. ^#^ indicates measurements carried out simultaneously.

November 2008–February 2009				
	PM_10_			PM_2.5_
	**BF station**	**GC station**	**DB station ^#^**	**DB station ^#^**
N	*95*	*108*	*105*	*40*
Mean	16	39	42	29
Std.Dev.	7	11	11	6
Median	15	39	43	30
Min	8	16	13	13
Max	44	69	74	40
Q_10_	10	26	28	22
Q_25_	12	32	35	25
Q_75_	17	46	49	33
Q_90_	27	53	55	37
***Saharan Dust Events***				
*N*	*7*	*7*	*8*	*4*
*Mean*	*130*	*158*	*133*	*78*
*Dev.St*	*89*	*81*	*59*	*28*
*Min*	*67*	*97*	*89*	*59*
*Max*	*261*	*276*	*220*	*120*

**Table 2:** Typical peaks of inorganic and organic molecules identified in filter samples during non-Saharan events and Saharan dust events.

Species	Frequency (cm^–1^) in This Study	Frequency (cm^–1^) from Literature	References
**Non-Saharan Events**			
SO_4_^2-^	603; 615; 670; 1100	608; 615; 670; 1100	[30,35,36,51,52,53]
CO_3_^2-^	713; 730; 877	713; 730; 873; 877	[53,57,58]
NO_3_^-^	825; 1356	825; 1318–1410; 1350	[30,58]
NH_4_^+^	1414	1414	[50]
C=C	1510–1596	1463–1511–1596	[59,61]
C-H	1460; 2850; 2920; 2950	2850–2920; 2800–3000	[30,31,34,58,59,60]
Water (OH)	1620; 3200–3400; 3620	1620; 3200–3400; 3620	[34,53,62]
Al-O-Si	540	540	[57,58]
Si-O	1030	1030	[30,62]
C = O	1720	1720; 1722	[34,58]
**Saharan Dust Events**			
O-Si-O	423; 463; 520	426; 468; 525	[63]
SO_4_^2-^	603; 615; 670; 1110	608; 615; 670; 1100	[30,60]
CO_3_^2-^	713; 730; 780; 877;1433	713; 730; 873; 877	[58,62]
Al-O-Si	750	750	[63,64]
Al-Al-OH	912	910	[62,65]
NO_3_^-^	825; 1356	825; 1318–1410; 1350	[30,32,58]
NH_4_^+^	1414	1414	[50]
C = C	1510–1596	1463–1511–1596	[59,61]
C-H	1460; 2800–3000	1460; 2850–2920; 2800–3000	[30,31,34,58,59]
Water (OH)	688; 1620; 1685; 3260–3400; 3620; 3669; 3695	688; 1620; 3200–3400; 3620, 3669; 3695	[34,62,63,66]
Si-O	1010; 1032	1010; 1030; 1031	[30,62,67]

**Table 3:** Soluble ion concentrations. Data expressed in µg/m^3^. nss, non-sea salt; ∑TP, total mass of ions; TPM, total particulate matter (µg/m^3^).

	PM_10_		PM_2.5_
	BF station	GC station	DB station
**F^-^**	0.15	0.15	0.17
**Cl^-^**	1.19	1.70	0.64
**NO_3_^-^**	2.30	4.13	2.91
**SO_4_^2-^**	2.69	2.26	2.49
**Na^+^**	1.14	1.47	0.74
**K^+^**	0.23	0.27	0.23
**Mg^2+^**	0.20	0.24	0.09
**Ca^2+^**	0.78	1.60	1.33
**NH_4_^+^**	0.96	1.32	1.52
**nssSO_4_^2-^**	2.44	2.05	2.10
**ΣTM**	9.64	13.1	10.1
**TPM**	19.5	37.0	29.3
